# Effect Biomarkers of the Widespread Antimicrobial Triclosan in a Marine Model Diatom

**DOI:** 10.3390/antiox11081442

**Published:** 2022-07-25

**Authors:** Bernardo Duarte, Eduardo Feijão, Ricardo Cruz de Carvalho, Ana Rita Matos, Maria Teresa Cabrita, Sara C. Novais, Ariana Moutinho, Marco F. L. Lemos, João Carlos Marques, Isabel Caçador, Patrick Reis-Santos, Vanessa F. Fonseca

**Affiliations:** 1 MARE—Marine and Environmental Sciences Centre & ARNET—Aquatic Research Infrastructure Network Associated Laboratory, Faculdade de Ciências da Universidade de Lisboa, Campo Grande, 1749-016 Lisbon, Portugal; emfeijao@fc.ul.pt (E.F.); rfcruz@fc.ul.pt (R.C.d.C.); micacador@fc.ul.pt (I.C.); vffonseca@fc.ul.pt (V.F.F.); 2 Departamento de Biologia Vegetal, Faculdade de Ciências da Universidade de Lisboa, Campo Grande, 1749-016 Lisbon, Portugal; armatos@fc.ul.pt; 3 cE3c, Centre for Ecology, Evolution and Environmental Changes, Faculdade de Ciências, Universidade de Lisboa, Campo Grande, Edifício C2, Piso 5, 1749-016 Lisboa, Portugal; 4 Plant Functional Genomics Group, BioISI—Biosystems and Integrative Sciences Institute, Departamento de Biologia Vegetal, Faculdade de Ciências da Universidade de Lisboa, Campo Grande, 1749-016 Lisbon, Portugal; 5 Centro de Estudos Geográficos (CEG), Instituto de Geografia e Ordenamento do Território (IGOT), Universidade de Lisboa, Rua Branca Edmée Marques, 1600-276 Lisboa, Portugal; tcabrita@campus.ul.pt; 6 MARE—Marine and Environmental Sciences Centre & ARNET—Aquatic Research Infrastructure Network Associated Laboratory, ESTM, Politécnico de Leiria, 2520-630 Peniche, Portugal; sara.novais@ipleiria.pt (S.C.N.); ariana.moutinho@ipleiria.pt (A.M.); marco.lemos@ipleiria.pt (M.F.L.L.); 7 MARE—Marine and Environmental Sciences Centre & ARNET—Aquatic Research Infrastructure Network Associated Laboratory, Department of Life Sciences, University of Coimbra, 3000 Coimbra, Portugal; jcmimar@ci.uc.pt; 8 Southern Seas Ecology Laboratories, School of Biological Sciences, The University of Adelaide, Adelaide, SA 5005, Australia; patrick.santos@adelaide.edu.au; 9 Departamento de Biologia Animal, Faculdade de Ciências da Universidade de Lisboa, Campo Grande, 1749-016 Lisbon, Portugal

**Keywords:** antimicrobials, ecotoxicology, energy metabolism, photobiology, primary producers

## Abstract

The present-day COVID-19 pandemic has led to the increasing daily use of antimicrobials worldwide. Triclosan is a manmade disinfectant chemical used in several consumer healthcare products, and thus frequently detected in surface waters. In the present work, we aimed to evaluate the effect of triclosan on diatom cell photophysiology, fatty acid profiles, and oxidative stress biomarkers, using the diatom *Phaeodactylum tricornutum* as a model organism. Several photochemical effects were observed, such as the lower ability of the photosystems to efficiently trap light energy. A severe depletion of fucoxanthin under triclosan application was also evident, pointing to potential use of carotenoid as reactive oxygen species scavengers. It was also observed an evident favouring of the peroxidase activity to detriment of the SOD activity, indicating that superoxide anion is not efficiently metabolized. High triclosan exposure induced high cellular energy allocation, directly linked with an increase in the energy assigned to vital functions, enabling cells to maintain the growth rates upon triclosan exposure. Oxidative stress traits were found to be the most efficient biomarkers as promising tools for triclosan ecotoxicological assessments. Overall, the increasing use of triclosan will lead to significant effects on the diatom photochemical and oxidative stress levels, compromising key roles of diatoms in the marine system.

## 1. Introduction

Antibiotics, hormones, pharmaceutical drugs, disinfectants, biocides, and UV filters, among others, can considered as pharmaceuticals and personal care products (PPCPs), a wide category of emerging contaminants [1]. Indeed, their increasing popularity has led to increased concern in recent years due to their presence in waters and consequent impacts on aquatic organisms [2]. Due to deficient elimination in wastewater treatment plants (WWTPs), PPCPs in household, industrial, and hospital disposal effluents enter the aquatic environments [3,4]. Their often low biodegradability rate and evaporation at normal temperature and pressure enhance PPCPs bioaccumulation potential and environmental presence, resulting in their detection even in remote locations such as Antarctica [5]. Several PPCPs are known to induce mild to severe effects in aquatic organisms, from fish to primary producers [6,7,8,9]. Therefore, the assessment of the impact of each emerging compound is key to an efficient and realistic environmental risk assessment [10].

Triclosan (5-chloro-2-(2,4-dichlorophenoxy)phenol) is a synthetic antimicrobial compound widely used as an antiseptic in a multiplicity of healthcare products. Triclosan is frequently detected in surface water globally at concentrations ranging from 0.011 to 2.7 μg/L, with untreated effluents exhibiting average values of 10 μg/L [11,12,13,14,15]. Triclosan has an octanol-water partition coefficient (log K_ow_) value of 4.8 at pH 7.5 [16] and its hydrophobicity increases its bioaccumulation potential and trophic transfer through the food web [17]. The effects of this PPCP have been widely studied in several aquatic organisms, mostly in terms of growth inhibition, with some lack of information regarding the physiological effects of triclosan in these organisms [18]. The EC_50_-96h values vary from 0.53 to 800 µg/L for microalgae, while for aquatic invertebrates the LC_50_-96h has been assessed to range from 184.7 to 3000 µg/L [18,19,20]. Nevertheless, most of these studies focused only on freshwater biota and on the survival or mortality of the organisms, without focusing on its physiological impacts and biomarker assessment. Triclosan is known to impair lipid synthesis by hindering enoyl-acyl carrier protein reductase (ENR), inducing cell membrane deterioration, leading to its permeabilization [21,22,23]. This is the basis of its antimicrobial characteristic, leading to cell disruption and consequent microorganism elimination [23].

Marine diatoms compose the large majority of estuarine and oceanic phytoplankton, and thus exposure to PPCPs may have severe implications at the ecosystem level [24,25]. Diatoms are cornerstones of marine food webs [26], being responsible for about 20% of the global primary production [27], being a major marine carbon sink and key oxygen-production source, essential for supporting marine heterotrophs [28]. *Phaeodactylum tricornutum* is a model marine diatom commonly used in stress physiology and ecotoxicological studies (e.g., thermal stress [29,30], metal toxicity [31,32,33], nutrient stress [34], or emerging contaminants [6,8,9]). Common physiological and biochemical traits evaluated in ecotoxicological trials using this model organism involve photobiological feedback [31,35], oxidative stress biomarkers [36], and fatty acids profiling [29,34,35]. These cellular features have high resolution and efficiency in disentangling the mechanisms of action of emerging contaminants, namely human-targeted PPCPs. Furthermore, diatoms produce essential fatty acids (EFAs), such as the omega 6 (ω-6) and omega 3 (ω-3) linolenic acids, Thus, any change either at the photochemical or at the biochemical levels, due to the exposure to any contaminant has the potential not only to provide efficient biomarkers, but also ecologically relevant biomarkers with implications at the system level. The joint use of non-invasive phenotyping techniques and conventional biochemical tools has been demonstrated to be an effective methodology in *P. tricornutum* ecotoxicological studies, delivering key data concerning the contaminants’ mode of action and correspondent cellular effects [31,35].

Considering this, the present work aims to evaluate triclosan mediated effects on the diatom species *P. tricornutum*. For this, *P. tricornutum* growth, energy and fatty acid metabolism, and oxidative stress are addressed to unravel this compound’s mode of action in diatoms, and the potential of these traits to be used as biomarkers of exposure to triclosan.

## 2. Materials and Methods

### 2.1. Experimental Setup

For the present work, a monoclonal *P. tricornutum* Bohlin (Bacillariophyceae) (strain IO 108-01, Instituto Português do Mar e da Atmosfera (IPMA)) axenic cell culture was kept under asexual reproduction conditions in f/2 medium [37] was used. The axenic state of the cultures is ensured throughout periodic visual inspection under the microscope. To perform the triclosan exposure trials, cultures were kept under controlled conditions as previously detailed [29] (Supplementary Figure S1). In short, cultures were placed in a phytoclimatic chamber (Temperature = 18 °C, 14/10 h day/night photoperiod, maximum PAR = 80 μmol photons m^−2^ s^−1^ (RGB 1:1:1,) programmed in a sinusoidal function to mimic sunrise and sunset, and light intensity at noon). Exposure was implemented according to the Organization for Economic Cooperation and Development (OECD) guidelines for algae assays [38], with minor adaptations. Initial cell density was set to 2.7 × 10^5^ cells mL^−1^, found to be adequate for microalgae cells with comparable dimensions to *P. tricornutum*. Aeration with ambient air was provided as the main carbon source. A 1 mg/L triclosan solution was prepared by dissolving triclosan (Sigma-Aldrich PHR1338 Certified Reference Material) in f/2 medium. After 48 h acclimation, cultures were added with adequate triclosan stock solution volumes to attain target final concentrations (0, 0.1, 1, 10, 50 and 100 μg/L triclosan). Target concentrations were chosen targeting to cover a concentration range mimicking not only realistic environmental concentrations presented in the literature [11,12,13], but also higher concentrations expectably illustrative of the levels linked with the rising biocide use under the present pandemic scenario [39,40]. Due to the fast growth rates of this diatom, the exposure time was reduced to 48 h, to avoid artefacts due to the ageing of the cells observed at 72 h, when the cultures already are in the stationary phase [29]. Aseptic conditions were ensured by performing all manipulations within a laminar flow hood chamber.

*Phaeodactylum tricornutum* cell density was monitored using a Neubauer improved counting chamber under an Olympus BX50 (Tokyo, Japan) inverted microscope (magnification = 400×). Mean specific growth rate (*SGR*) per day, doubling time (*d*, in days), and the number of divisions per day (*M*) were calculated according to [41]:

$$d=ln\frac{2}{SGR}\;\mathrm{and}\;M=\frac{1}{d}$$

At the end of the 48 h of the exposure period, cells were analysed photochemically as described below (Section 2.2), centrifuged (4000× *g* for 15 min at 4 °C), harvested for biochemical analysis, and flash-frozen in liquid nitrogen and stored at −80 °C. All tested conditions involved three biological replicates for each analysis, constituting a total of 18 experimental units.

### 2.2. Pulse Amplitude Modulated (PAM) Chlorophyll a Fluorometry

Before cell harvesting, 1 mL of each replicate culture was used for photochemical chlorophyll *a* fluorescence measurements, using pulse amplitude modulated (PAM) fluorometry (FluorPen FP100, Photo System Instruments, Brno, Czech Republic). Culture subsamples for photochemical assessment were dark-adapted for 15 min and chlorophyll transient light curves were produced using the pre-programmed OJIP protocol, according to [7]. Derived parameters are presented in Table 1.

### 2.3. Pigment Profiles

Cell pellets were homogenized with pure acetone, sonicated to ensure cell disruption and extracted overnight at −20 °C to avoid pigment degradation [8]. After extraction, samples were centrifuged at 4000× *g* for 15 min at 4 °C, and the supernatant was scanned using a dual-beam UV-1603 spectrophotometer (Shimadzu, Kyoto, Japan). Absorbance spectral data (350 nm to 750 nm, 0.5 nm steps) were explored using a Gauss-Peak Spectra (GPS) fitting library, using SigmaPlot Software, and pigments were quantified using the algorithm developed by [42]. Xanthophyll de-epoxidation state (DES) was calculated as the ratio between diatoxanthin and diadinoxanthin.

### 2.4. Fatty Acid Profiles

Cell pellets were directly trans-esterified, in methanol sulfuric acid (97.5:2.5, *v*/*v*), at 70 °C for 60 min and the generated fatty acids methyl esters (FAMEs) extracted using petroleum ether and dried under an N_2_ stream in a dry bath at 30 °C [7,8]. After reconstitution with hexane, FAMEs were analysed in a gas chromatograph (Varian 430-GC gas chromatograph, Varian, Inc., Palo Alto, CA, USA) under previously described chromatographic conditions [7,8]. Fatty acid identification was achieved by similarity with retention times obtained from attained from a standard collection (Sigma-Aldrich). Chromatograms were analysed by the peak surface method, using the Galaxy software. Pentadecanoic acid (C15:0) was used as internal standard. The double bond index (DBI) was calculated as:

$$\mathrm{DBI}=\frac{2\times \left(\%\;\mathrm{monoenes}+2\times \%\;\mathrm{dienes}+3\times \%\;\mathrm{trienes}+4\times \%\;\mathrm{tetraenes}+5\times \%\;\mathrm{pentaenes}\right)}{100}$$

### 2.5. Oxidative Stress Biomarkers

Soluble protein was extracted cell pellets by sonication (1 min) with 50 mM sodium phosphate buffer (pH 7.6, supplemented with 0.1 mM Na-EDTA). After centrifugation (10,000× *g* for 10 min at 4 °C), the supernatant was collected and its protein concentration was determined according to [43]. Catalase (CAT), ascorbate peroxidase (APx) and superoxide dismutase (SOD) activities were analysed by spectrophotometry, in the protein extract and using specific substrates as previously described [7,36]. The oxidative ratio was calculated as:

$$\mathrm{Oxidative}\;\mathrm{ratio}=\frac{\mathrm{SOD}}{\mathrm{CAT}+\mathrm{APx}}$$

where SOD, CAT, and APx are the activity values of superoxide dismutase, catalase, and ascorbate peroxidase activities, respectively.

Lipid peroxidation products (here evaluated through the thiobarbituric acid reactive substances, TBARS) were analysed spectrophotometrically [7,36], using trichloroacetic acid extraction before the reaction with thiobarbituric acid.

### 2.6. Cell Energy Allocation and Mitochondrial Metabolism

Cell pellets were extracted with 1 mL of ultra-pure water and disrupted by ultra-sonification. Supernatants were used to evaluate the lipid, carbohydrate, and protein contents as well as electron transport system (ETS) activity. All assays were performed at −25 °C with Milli-Q water as a reaction blank in all assays. All analysis were made by spectrophotometric means (Synergy H1 hybrid multimode microplate reader, Biotek^®^ Instrument, Winooski, VT, USA). Total lipid, protein, and carbohydrate extraction and analysis were performed according to [44], with minor modifications [7,8,45]. Total protein, carbohydrate, and lipid contents were converted into energetic equivalents for available energy (Ea) quantification (respective combustion energies: 17,500 mJ mg^−1^ carbohydrates, 24,000 mJ mg^−1^ protein and 39,500 mJ mg^−1^ lipid) [45]. The mitochondrial electron transport system (ETS) activity was analysed according to [46] with the modifications described by [7,8,45]. The calculated oxygen consumption was converted using the specific oxyenthalpic equivalents for an average lipid, protein, and carbohydrate mixture of 480 kJ mol^−1^ O_2_ into energetic equivalents [47]. Cellular energy allocation (CEA) values were determined based on Ea and ETS activity [48].

### 2.7. Statistical Analysis

Spearman correlation coefficients and significance were assessed using the corrplot package [49]. Barplots were plotted using the ggplot2 package [50]. Non-parametric Kruskal–Wallis with Bonferroni posthoc tests were performed using the agricolae package [51]. Canonical analysis of principal coordinates (CAP) was used to evaluate the ability to successfully classify individuals according to the triclosan exposure concentrations using each of the considered biochemical and biophysical traits [26,37,38], using Primer 6 software [52]. All other analyses were run in R-Studio 1.4.1717.

## 3. Results

### 3.1. Diatom Cell Growth

**Figure 1:**
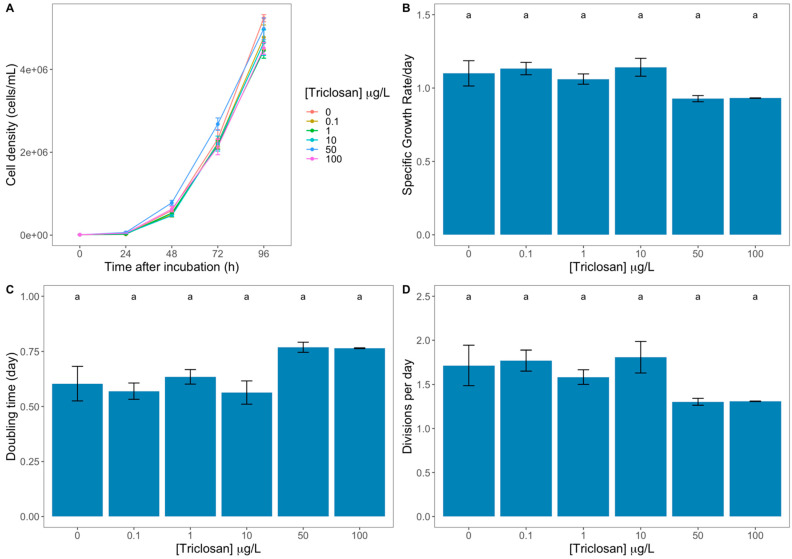
Triclosan exposed *Phaeodactylum tricornutum* cultures growth curves (**A**), specific growth rate (**B**), doubling time (**C**), and the number of divisions per day on a cellular basis (**D**) (average ± standard error, n = 3; different letters denote significant differences between triclosan treatments at *p* < 0.05).

From the evaluation of the growth curves and derived parameters (Figure 1A–D), there is no apparent impact of the tested triclosan concentrations on cell growth. Nevertheless, when observing the correlation between triclosan dosage and the growth traits, some significant correlations arise (Supplementary Figure S2A). Diatom specific growth rate (SGR) and the number of cell divisions per day (M) showed a significant and inverse correlation with the applied triclosan concentration. Oppositely, cell doubling time (D) showed a strong direct and significant correlation with the triclosan concentration to which the cells were subjected.

### 3.2. Photochemical Traits

**Figure 2:**
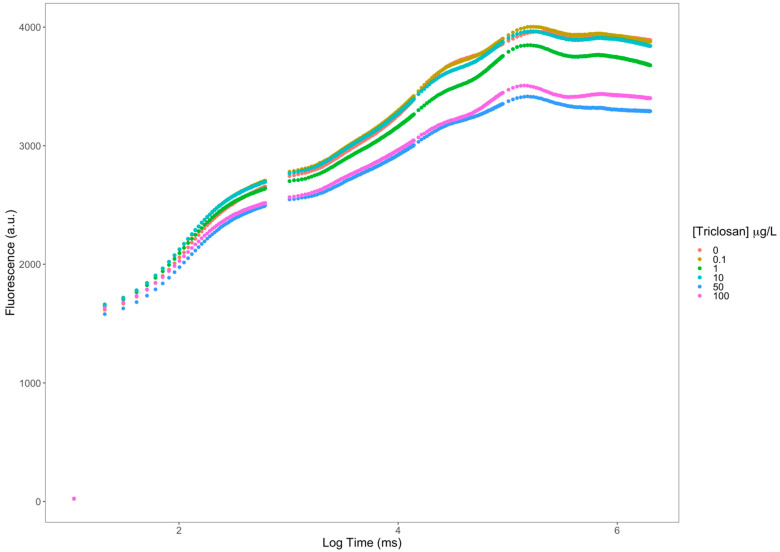
Kautsky plot curves (fluorescence in arbitrary units (a.u.)) from *Phaeodactylum tricornutum* triclosan-exposed cultures (average; n = 3 per treatment).

Observing the Kautsky induction curves (Figure 2), it is evident that the exposure to increasing triclosan concentrations led to effects over the several steps of the photochemical process. Not only is the fluorescence intensity lower in the samples exposed to higher triclosan concentrations, but it is also possible to observe some changes in the curves’ shape, which inevitably impacts the values of the derived variables.

**Figure 3:**
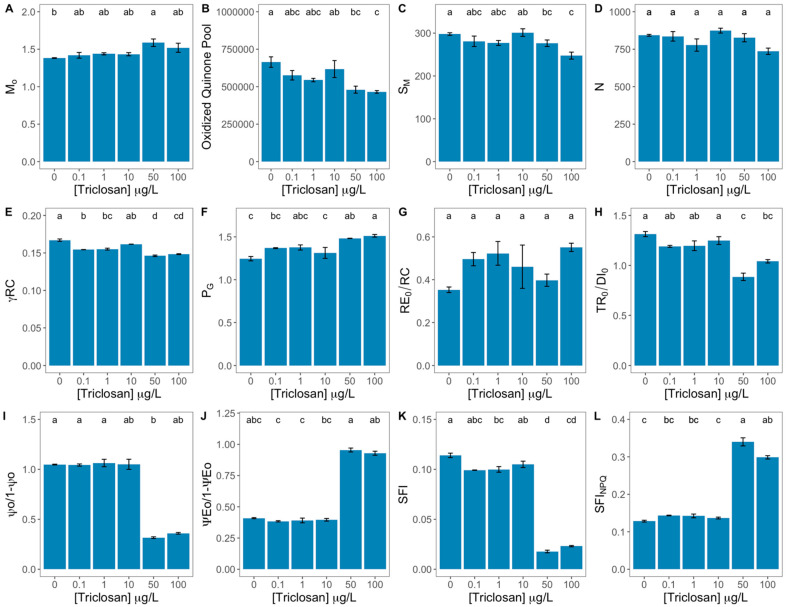
Triclosan-exposed *Phaeodactylum tricornutum* cultures photochemical traits [quinone A reduction rate (M_o_, **A**), oxidized quinone pool size (**B**), energy needed to close all reaction centres (S_M_, **C**), reaction centre turnover rate (N, **D**), the probability that a chlorophyll molecule acts as a reaction centre (γRC, **E**), grouping probability (P_G_, **F**), electron transport from PQH_2_ to the reduction of PSI end electron acceptors (RE_0_/RC, **G**), contribution or partial performance due to the light (TR_0_/DI_0_, **H**) and dark (ψ_0_/1 − ψ_0_, **I**), the equilibrium constant for the redox reactions between PS II and PS I (ψE_0_/1 − ψE_0_, **J**), structural and functional indexes of the photochemical (SFI, **K**) and non-photochemical (SFI_NPQ_, **L**) reactions of the primary photochemistry] (average ± standard error, n = 3; different letters denote significant differences between triclosan treatments at *p* < 0.05).

Observing the quinone A reduction rate (Figure 3A), this parameter was found to be lower in the control samples and significantly higher in the samples exposed to the 60 μg/L triclosan concentrations. A significant direct correlation was found between this variable and the exogenous triclosan dose (Supplementary Figure S2B). Regarding the size of the oxidized quinone pool of the electron transport chain (ETC), this parameter showed significantly lower values upon the exposure to 50 and 100 μg/L (Figure 3B), showing a significant inverse correlation with the exogenous dose of this anti-microbial (Supplementary Figure S2). The same tendency was observed in the energy needed to close all reaction centres (Supplementary Figure S2), as this value was significantly lower in the diatom cells exposed to the higher triclosan concentrations (Figure 3C). Triclosan tested concentrations did not affect the reaction centre turnover rate (Figure 3D) in the exposed diatoms. Regarding the probability that a chlorophyll molecule acts as a reaction centre (Figure 3E), the same effect was observed upon the application to the highest triclosan concentrations, being this decrease significantly correlated with the increasing triclosan levels tested (Supplementary Figure S2). Analysing the data referent to the PSII antennae dysconnectivity (here evaluated through the grouping probability, Figure 3F), it was possible to observe a significant increase in the cells exposed to 50 and 100 μg/L triclosan, which was directly and significantly correlated with the triclosan exposure dose (Supplementary Figure S2). Nevertheless, the electron transport from PQH_2_ to the reduction of PSI end electron acceptors was not affected by any of the tested triclosan concentrations (Figure 3G). The contribution or partial performance due to the light reactions (Figure 3H) was found to be significantly reduced with exposure to 50 and 100 μg/L triclosan, while the contribution or partial performance due to the dark reactions (Figure 3I) was only substantially decreased with the application of 50 μg/L triclosan. Both these parameters showed a strong and significant inverse correlation with the exogenous triclosan dose applied (Supplementary Figure S2). Evaluating the equilibrium constant for the redox reactions between PS II and PS I (Figure 3J) there was a strong direct correlation with the triclosan concentration to which the cells were subjected (Supplementary Figure S2), favouring the PSII redox activity. Analyzing two structural and functional photochemical indexes it is possible to observe that under the highest triclosan doses tested, the SFI suffers significant decreases (Figure 3K), while its non-photochemical congener (SFI_NPQ_, Figure 3L) showed the opposite trend. This was confirmed by the strong direct and inverse correlations verified, respectively, between the SFI and the SFI_NPQ_ and the triclosan exogenous dose.

**Figure 4:**
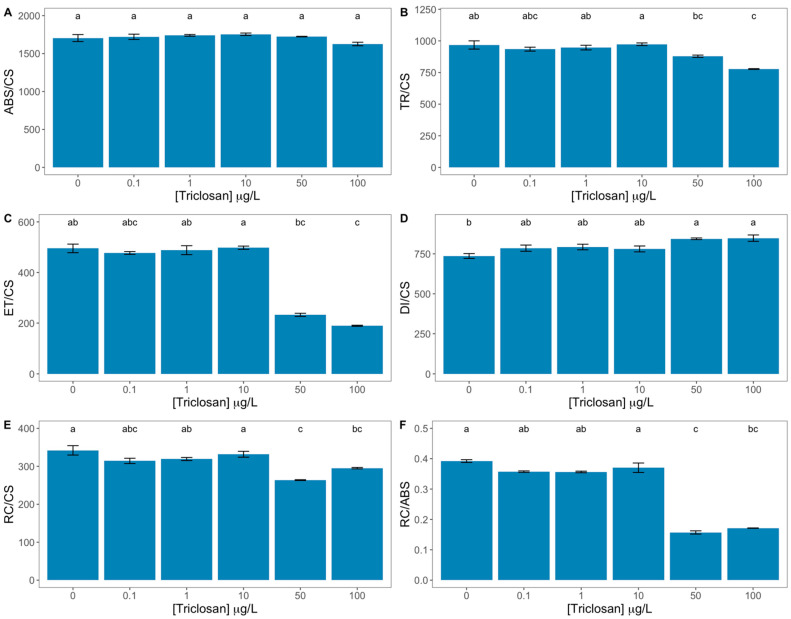
Triclosan-exposed *Phaeodactylum tricornutum* cultures phenomological energy fluxes [absorbed (ABS/CS, **A**), trapped (TR/CS, **B**), transported (ET/CS, **C**) and dissipated (DI/CS, **D**) energy fluxes], number of available reaction centres per cross-section (RC/CS, **E**) and reaction centre II density within the antenna chlorophyll bed of PS II (RC/ABS, **F**) (average ± standard error, n = 3; different letters denote significant differences between triclosan treatments at *p* < 0.05).

Differences in energy fluxes that rule the photochemical energy transduction pathway were also found (Figure 4). While no effects were detected in the absorbed energy flux (Figure 4A), exposure to 50 and 100 μg/L triclosan led to significant reductions in the trapped and transported energy fluxes (Figure 4B,C respectively), as well as in the number of available reaction centres per cross-section (Figure 4E) and of the reaction centre II density within the antenna chlorophyll bed of PS II (Figure 4F). Additionally, these variables also showed strong inverse correlations with the exogenous triclosan dose tested (Supplementary Figure S2B). On the other hand, the dissipated energy flux showed a significant increase in the cells exposed to the highest triclosan doses (Figure 4D), accompanied by a significant and direct correlation with the triclosan exogenous dose (Supplementary Figure S2B).

### 3.3. Pigment Composition

**Figure 5:**
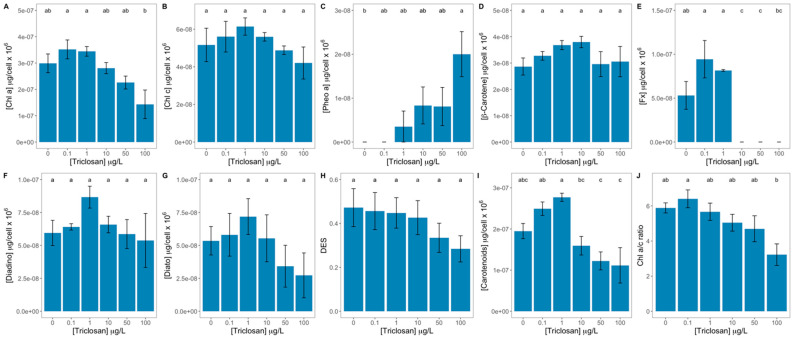
Triclosan-exposed *Phaeodactylum tricornutum* cultures pigment concentrations [chlorophyll *a* (Chl a, **A**), chlorophyll *c* (Chl *c*, **B**), pheophytin *a* (Pheo *a*, **C**), β-carotene (**D**), fucoxanthin (Fx, **E**), diadinoxanthin (Diadino, **F**), diatoxanthin (Diato, **G**)], de-epoxidation index (DES, **H**), total carotenoid concentration (**I**), and chlorophyll *a* to *c* ratio (**J**) (average ± standard error, n = 3; different letters denote significant differences between triclosan treatments at *p* < 0.05).

The exposure to the highest triclosan concentration tested led to a significant reduction of the diatom cell’s chlorophyll *a* concentration (Figure 5A), concomitant with an also significant increase in pheophytin *a* concentration (Figure 5C). Both these pigments showed a significant relationship with the exogenous triclosan dose applied (Supplementary Figure S2C). Although no significant differences could be detected in the chlorophyll *c* concentration between the different triclosan concentrations tested (Figure 5B), a significant inverse correlation could be observed between this pigment and the anti-microbial concentration applied (Supplementary Figure S2C). Regarding β-carotene, triclosan had no significant impact on this pigment concentration (Figure 5D). Contrastingly, fucoxanthin was severely impacted by triclosan (Figure 5E) being depleted in the cells exposed to 10, 50, and 100 μg/L triclosan. This effect was found to be strongly correlated with the anti-microbial dose applied (Supplementary Figure S2C). None of the pigments involved in the diatom xanthophyll cycle (diadinoxanthin and diatoxanthin) showed any significant differences along the triclosan gradient tested (Figure 5F,G). This also led to no alterations at the de-epoxidation state level (DES, Figure 5H). Nevertheless, the three variables presented a significant inverse correlation with the triclosan exogenous dose tested (Supplementary Figure S2C). Total carotenoid content was significantly reduced in diatom cells exposed to 50 and 100 μg/L triclosan, especially when compared to the cells exposed to low (0.1 and 1 μg/L) triclosan concentrations (Figure 5I), strongly correlated to dose applied. The chlorophyll *a*/*c* ratio was found significantly reduced in the cells exposed to 100 μg/L triclosan when compared with the cultures exposed to 0.1 μg/L (Figure 5J). Nevertheless, this variable also showed an a strong and significant inverse correlation with the exogenous anti-microbial dose applied (Supplementary Figure S2C).

### 3.4. Fatty Acid Profile

**Figure 6:**
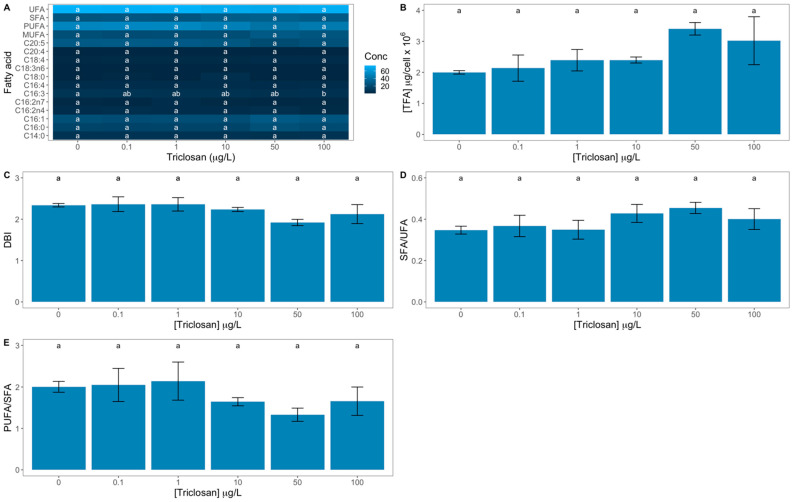
Triclosan-exposed *Phaeodactylum tricornutum* cultures fatty acid profiles and respective saturation classes relative concentrations [myristic acid (C14:0), palmitic acid (16:0), palmitoleic acid (16:1), hexadecadienoic acid (C16:2n4), palmitolenic acid (C16:2n7), palmitolinolenic acid (C16:3), palmitidonic acid (C16:4), stearic acid (C18:0), γ-linolenic acid (C18:3), stearidonic acid (C18:4), arachidonic acid (C20:4), eicosapentaenoic acid (C20:5), monounsaturated (MUFA), Polyunsaturated (PUFA), saturated (SFA) and unsaturated (UFA) fatty acids, **A**], total fatty acid content (TFA, **B**), double-bound index (DBI, **C**), saturated to unsaturated fatty acids ratio (SFA/UFA, **D**), and polyunsaturated to saturated fatty acid ratio (PUFA/SFA, **E**) (average ± standard error, n = 3; different letters denote significant differences between triclosan treatments at *p* < 0.05).

Comparing the relative concentrations of each fatty acid at each tested concentration, only the palmitolinolenic acid (C16:3) showed a significant decrease upon exposure to 100 μg/L triclosan (Figure 6A). Nevertheless, subtle non-significant increases and decreases contributed to several significant direct correlations between fatty acid traits and the exogenous triclosan dose applied, namely with the palmitic acid (C16:0), palmitoleic acid (C16:1) and stearidonic acid (C18:4) (Supplementary Figure S2D). Also, the monounsaturated fatty acids relative concentration and the total fatty acid concentration presented a similar trend, as well as the SFA/UFA ratio (Supplementary Figure S2D). Oppositely, the palmitolinolenic (C16:3) acid and eicosapentaenoic acid (C20:5) showed an inverse and significant correlation with the tested triclosan doses. The same could be verified for the polyunsaturated fatty acid relative concentration (PUFA), PUFA/SFA ratio, and DBI (Supplementary Figure S2D). Considering the cell total fatty acid content, as well as the saturation indexes, evaluated, no significant differences were found between the diatom cells exposed to different triclosan concentrations.

### 3.5. Cellular Bioenergetics

**Figure 7:**
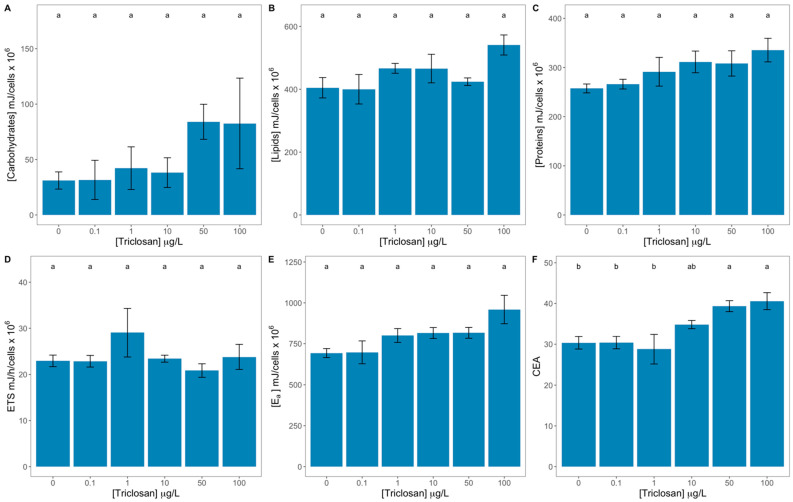
Triclosan-exposed *Phaeodactylum tricornutum* cultures energetic traits [carbohydrates (**A**), lipids (**B**), proteins (**C**), electron transport system (ETS, **D**), available energy (E_a_, **E**), and cellular energy allocation (CEA, **F**)] (average ± standard error, n = 3; different letters denote significant differences between triclosan treatments at *p* < 0.05).

Triclosan did not significantly affect the content of carbohydrates (Figure 7A), lipids (Figure 7B), and proteins (Figure 7C) nor the electron transport system activity (Figure 7D) or the overall available energy (Figure 7E). Regarding the cellular energy allocation (CEA), this was found to be increased in the cultures exposed to 50 and 100 μg/L triclosan when compared to the remaining treatments (Figure 7F). If the linear relationships between the exogenous triclosan dose applied and these traits are observed, it is possible to observe that CEA displays a significant positive correlation with the triclosan concentration to which the cells were subjected (Supplementary Figure S1E).

### 3.6. Oxidative Stress Biomarkers

**Figure 8:**
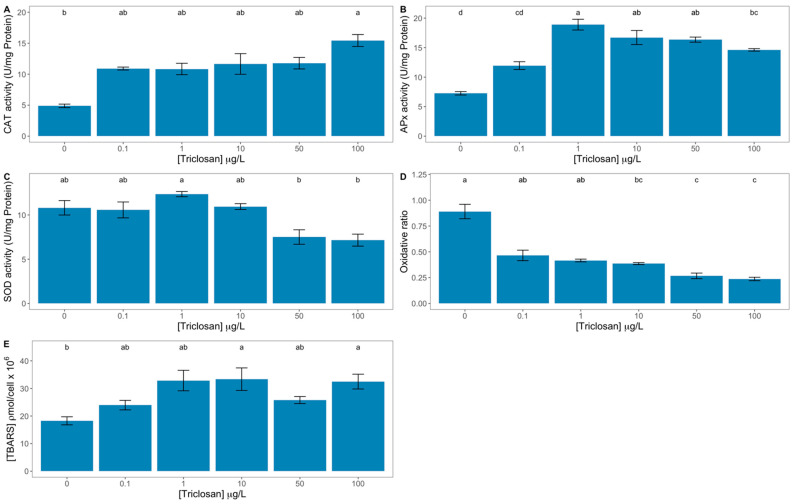
Triclosan-exposed *Phaeodactylum tricornutum* cultures oxidative stress biomarkers [catalase activity (CAT, **A**), ascorbate peroxidase activity (APx, **B**), superoxide dismutase (SOD, **C**), and thiobarbituric reactive substances (TBARS, **E**)] and oxidative ratio (**D**) (average ± standard error, n = 3; different letters denote significant differences between triclosan treatments at *p* < 0.05).

Concerning oxidative stress biomarkers, several effects were detected (Figure 8). Catalase (Figure 8A) was significantly enhanced in the samples exposed to 100 μg/L triclosan. This increase was found to be significantly correlated with the triclosan exogenous dose applied (Supplementary Figure S2F). Ascorbate peroxidase was significantly increased in the diatom cells exposed to triclosan concentrations higher than 1 μg/L triclosan (Figure 8B). Superoxide dismutase was significantly inhibited under 50 and 100 μg/L triclosan concentration when compared to the high activity value observed for the cells exposed to 1 μg/L (Figure 8C), with this decrease being significantly correlated with the anti-microbial dose applied (Supplementary Figure S2F). Analysing the integrative oxidative ratio (Figure 8D) disclosed a significant decrease in this ratio upon exposure to triclosan concentrations above 10 μg/L. This variable also showed a significant inverse correlation with the anti-microbial dose applied (Supplementary Figure S2F). The membrane damage effects, here evaluated through the TBARS concentration, presented a significant rise in the cells exposed to 10 and 100 μg/L triclosan (Figure 8E), with this increase being significantly correlated with the anti-microbial concentration applied (Supplementary Figure S2F).

### 3.7. Biomarker Profiling

**Figure 9:**
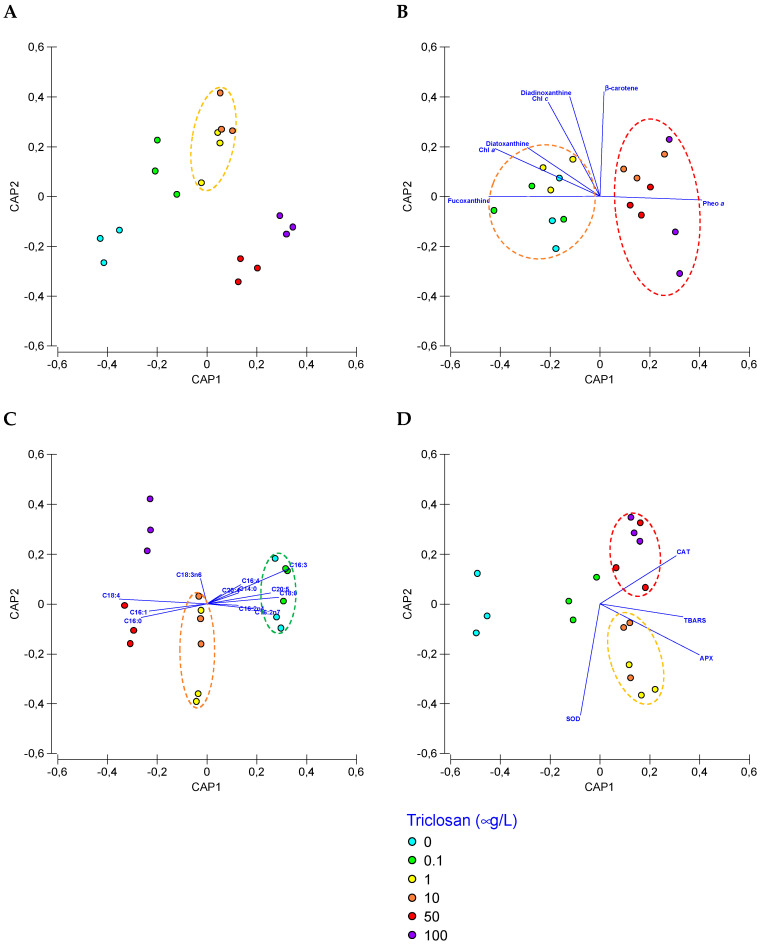
Canonical analysis of principal (CAP) components of the bio-optical data (**A**), pigment concentrations (**B**), fatty acids profiles (**C**), and oxidative stress biomarkers (**D**) obtained from the analysis of *Phaeodactylum tricornutum* cultures exposed to the different triclosan concentrations.

Analysing the different photochemical and biochemical traits in a multivariate approach allowed us to observe ways in which different trait datasets responded to the tested triclosan concentrations (Figure 9). None of the evaluated datasets was able to describe with 100% efficiency the triclosan concentrations to which the cells were exposed. Based on the photochemical Kautsky induction curve fluorescence values (Figure 9A) only 50% of the samples could be correctly classified, with high misclassifications at intermediate triclosan concentrations (1, 10, and 50 μg/L, 33.3% classification efficiency). The pigment and fatty acid profiles (Figure 9B,C, respectively) also showed low-resolution power in depicting exposure dose (38.9% and 22.2% respectively). Regarding oxidative stress (Figure 9D), the canonical analysis revealed a high classification efficiency (72.2%), highlighting the potential of these oxidative stress traits as biomarkers of exposure to triclosan, however still having difficulty in distinguishing among 10 μg/L and 0.1, 1, or 50 μg/L exposures.

## 4. Discussion

Triclosan is one of the most widespread anti-microbial agents used in a wide variety of consumer healthcare products, soaps, and plastics. Thus, its presence in the aquatic environment has been recorded frequently [11,14,15]. At the tested concentrations, triclosan did not induce any significant decrease in diatom growth, thus disabling the use of the typical growth inhibition tests as a biomarker of its effects [38]. Although in surface waters triclosan concentration has been found in the range of 0.011–2.7 μg/L, with maximum values measured found in untreated waters (10 μg/L) [11,12,13], it is important to stress that under the current COVID-19 pandemic or with the emergence of new microbial threats and consequent increase in the use of antimicrobial agents, the environmental concentration is expected to rise [39,40].

Previous reports [53,54] found that triclosan exposure suppressed molecular signalling pathways including porphyrin and chlorophyll metabolism, and photosynthesis was suppressed in green algae. In this work, triclosan exposure led to the reduction of the connectivity of the PS II antennae, essential for energy capture and transduction. These implications are most evident when observing the reduction in the trapped energy flux, which was severely reduced in the cells exposed to the two highest triclosan concentrations tested. This leads to an disruption of the energy transduction of the PSII to the ETC, increasing the likelihood that the cells undergo photoinhibition potentially damaging conditions, driven by excessive redox potential build-up within the photosystems, which can eventually lead to D1 protein destruction and inactivation of the PS II repair cycles [55]. Nevertheless, the high dissipated energy flux under exposure to higher triclosan concentrations indicates a deterrence of the PS II donor side stored energy from the photosystems, one of the most common counteractive measures toward permanent photo-inhibition [7,56,57]. Additionally, the reduction of PS II antennae RC centre density accompanied by an increase in the energy required to close all RCs, as well as in the RCs turnover rates, indicates a possible activation of another counteractive measure to avoid unnecessary photonic energy to be absorbed. This strategy was also previously observed in *P. tricornutum* cells exposed to propranolol [7]. This lack of efficiency in harvesting light results not only from impaired PS II antennae connectivity, but also from the reduced probability that a chlorophyll molecule can capture light energy, as highlighted by the changes observed in the chlorophyll content of the cells.

At high levels of exogenous triclosan, a severe reduction of the chlorophyll *a* cell content was detected with a concomitant increase in its degradation product pheophytin *a*, indicating that triclosan induced the degradation of this essential light-harvesting pigment (in opposition to a chlorophyll a biosynthesis inhibition) [58], which contributed to the low efficiency of the PS II. Another of the most evident effects was related to the structure and function of the chloroplast quinone electron transport chain, reduction of the oxidized quinone pool, and its redox turnover. This leads to an inhibition of the electron transport energy flux and consequent increase in the energy dissipation. This leads to a reduction of the energy arriving the PS I, disrupting the energy between both photosystems and impairing the reduction of PS I end acceptors [35]. Additionally, the impairment of the contribution of the dark reactions to primary photochemistry leads to a blockage of Calvin cycle substrate regeneration, downstream the PS I [59].

Cells can dissipate energy by biophysical (energy dissipation through heat and fluorescence) or biochemical (through de-epoxidation reaction in the xanthophyll cycle) means [56]. Triclosan did not induce the activation of the xanthophyll cycle, indicating that the excess energy is being diverted through heat and fluorescence, a common feature already reported for diatoms under propranolol exposure [7]. At the carotenoid level, there was also evidence of severe depletion of fucoxanthin under triclosan application, indicating a potential impairment its biosynthetic pathway downstream β-carotene [8], as this carotenoid is a key precursor in this pathway, and presents rather stable values. Compromised carotenoid production has also consequences at the oxidative stress level, as several carotenoids are also described to counteract lipid peroxidation and scavenge reactive oxygen species [8,60]. In metabolic terms, diatom cells exposed to high triclosan concentrations apparently preferred the use of carotenoid-based ROS (reactive oxygen species) quenchers such as β-carotene in opposition to the use of other carotenoid pigments, such as diadinoxanthin and diatoxanthin, key players for counteracting photoinhibition, that in this case was prevented by biophysical means. A reduction in fucoxanthin was also observed, leading to the inevitable reduction of the fucoxanthin chlorophyll *a*/*c*-binding protein (FCP), an exclusive light-harvesting and excitation energy transfer present in diatoms [61] and is related with observed the decoupling of the PS II antennae as well as with the reduced trapped energy flux in diatoms exposed to high triclosan concentrations.

Photochemical impairment leads to excessive redox potential accumulation and oxidative stress, with implications at the fatty acid composition level [30] as well as in terms of the generation of lipid peroxidation products [36]. Yet, no significant changes could be observed either in its fatty acid composition. Nevertheless, a significant increase in the lipid peroxidation products of the cells exposed to mild and high triclosan concentrations was detected. Observing the oxidative ratio, a promotion of the peroxidasic activity in detriment of the SOD activity [62] is evident, indicating a high generation of hydroxyl radicals by peroxidasic activity [63], which without iron-based Fenton reactions will not be efficiently quenched and prone to produce lipid hydroperoxides, thus leading to the lipid peroxidation products increase. This lack of SOD efficiency reinforces the role of carotenoid-ROS quenching mechanisms and the abovementioned carotenoid depletion.

A high amount of stored energetic substrates (lipids, proteins, and carbohydrates) could be observed alongside the triclosan gradient applied, without reduction of the mitochondrial electron transport rate (directly related to respiratory activity). This indicates mitochondrial function maintenance and increased storage of energy of the cells, resulting in an increased energy budgets (CEA) verified by increasing triclosan concentrations. According to previous studies [48], an increase in CEA and consequently in the net energy budget indicates that more energy is available to fundamental functions (e.g., growth), thus preventing growth reductions, despite the photochemical impairments observed.

Univariate analysis indicated parameters in isolation are not efficient biomarkers of Triclosan exposure. However, considering the metabolic interconnectivity of these parameters, integrated approaches not only highlight which metabolic pathways (photochemical, fatty acid, pigment, oxidative stress) provide the best set of biomarkers for triclosan exposure classification, but also insight regarding the parameters contributing most to dose-related effects. In this case, the oxidative stress biomarkers were the most suitable and efficient, following ecotoxicological evaluations of classical and emerging contaminants [36].

## 5. Conclusions

Although no signs of growth inhibition were detected, significant effects at the photochemical and oxidative stress levels were verified, which may compromise the key role of these organisms in the marine system. Available triclosan concentration data in the aquatic environment point to values below those with expectable adverse effects according to this study. Nevertheless, the pandemic scenario that has afflicted the world population has led to an increased use of antimicrobials, namely triclosan, and there is the potential that resulting higher concentrations of triclosan in the aquatic environments could induce effects on marine diatoms, as seen here. Importantly, the present work highlights how using oxidative stress biomarkers could be represent an important tool for future monitoring programs and ecotoxicological assessments regarding the presence and potential impacts of triclosan in marine environments.

## Figures and Tables

**Table 1:** Summary of the photochemical parameters used in the present work and their description.

OJIP-Test	Parameter Description
Area	Corresponds to the oxidized quinone pool size available for reduction and is a function of the area above the Kautsky plot
N	Reaction centre turnover rate
S_M_	Corresponds to the energy needed to close all reaction centres
M_o_	Quinone A reduction rate
γ_RC_	Probability that a chlorophyll molecule acts as a reaction centre.
P_G_	Grouping probability between the two PSII units
ABS/CS	Absorbed energy flux per cross-section
TR/CS	Trapped energy flux per cross-section
ET/CS	Electron transport energy flux per cross-section
DI/CS	Dissipated energy flux per cross-section
RC/CS	Number of available reaction centres per cross-section
RE_0_/RC	Electron transport from PQH_2_ to the reduction of PSI end electron acceptors
TR_0_/DI_0_	Contribution or partial performance due to the light reactions for primary photochemistry
ψ_o_/(1 − ψ_o_)	Contribution or partial performance due to the dark reactions for primary photochemistry
ψ_E0_/(1 − ψ_E0_)	Equilibrium constant for the redox reactions between PS II and PS I
RC/ABS	Reaction centre II density within the antenna chlorophyll bed of PS II
SFI	Structural and Functional Index of the photochemical reactions
SFI (NPQ)	Structural and Functional Index of the non-photochemical reactions

## Data Availability

Data available upon request.

## Supplementary Materials

The following supporting information can be downloaded at: https://www.mdpi.com/article/10.3390/antiox11081442/s1, Figure S1: Overview of the *Phaeodactylum tricornutum* cultivation units within the growth chamber; Figure S2: Spearman correlogram between *Phaeodactylum tricornutum* growth, photochemical, pigment, fatty acid, energetic and oxidative stress traits and the exogenous triclosan concentration applied (only significant correlations at *p* < 0.05 are shown; N = 3 per treatment).
