# Supplementary material for: Effect Biomarkers of the Widespread Antimicrobial Triclosan in a Marine Model Diatom
Source: Antioxidants (Basel). 2022 Jul 25;11(8):1442. doi: 10.3390/antiox11081442 (PMC9330214; doi:10.3390/antiox11081442)
Supplement: Supplementary file 1 [file antioxidants-11-01442-s001.zip › antioxidants-1831047-supplementary.pdf]

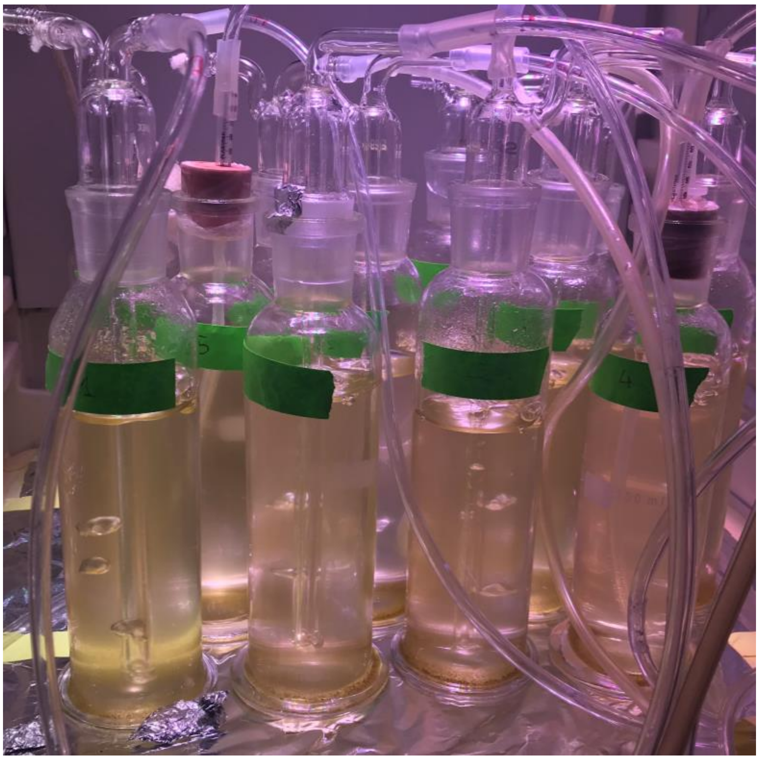

Supplementary Figure S1. Overview of the *Phaeodactylum tricornutum* cultivation units within the growth chamber.

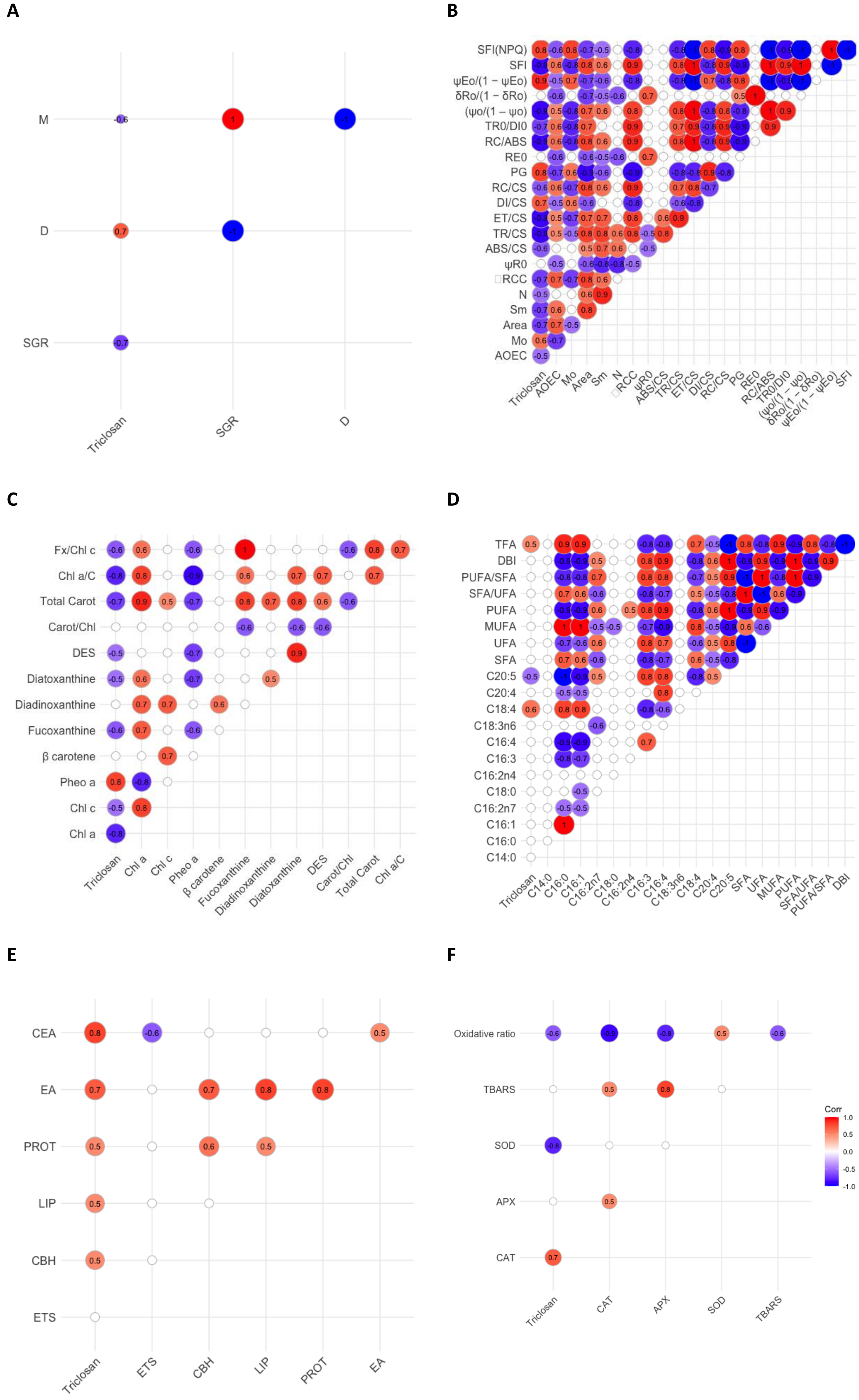
A
M
D
SGR
Triclosan
SGR
D
B
SFI(NPQ)
SFI
ψEo/(1 − ψEo)
δRo/(1 − δRo)
(ψo/(1 − ψo)
TR0/DI0
RC/ABS
RE0
PG
RC/CS
DI/CS
ET/CS
TR/CS
ABS/CS
ψR0
RCC
N
Sm
Area
Mo
AOEC
Triclosan
C
Fx/Chl c
Chl a/C
Total Carot
Carot/Chl
DES
Diatoxanthine
Diadinoxanthine
Fucoxanthine
β carotene
Pheo a
Chl c
Chl a
Triclosan
D
TFA
DBI
PUFA/SFA
SFA/UFA
PUFA
MUFA
UFA
SFA
C20:5
C20:4
C18:4
C18:3n6
C16:4
C16:3
C16:2n4
C18:0
C16:2n7
C16:1
C16:0
C14:0
Triclosan
E
CEA
EA
PROT
LIP
CBH
ETS
Triclosan
F
Oxidative ratio
TBARS
SOD
APX
CAT
Triclosan
Corr
1.0
0.5
0.0
-0.5
-1.0

Supplementary Figure S2. Spearman correlogram between *Phaeodactylum tricornutum* growth, photochemical, pigment, fatty acid, energetic and oxidative stress traits and the exogenous triclosan concentration applied (only significant correlations at $p < 0.05$ are shown; N = 3 per treatment).
